# Comparative Study of Algal Responses and Adaptation Capability to Ultraviolet Radiation with Different Nutrient Regimes

**DOI:** 10.3390/ijerph19095485

**Published:** 2022-04-30

**Authors:** Lingxiao Ren, Jing Huang, Keqiang Ding, Yi Wang, Yangyang Yang, Lijuan Zhang, Haoyu Wu

**Affiliations:** 1 School of Environmental Engineering, Nanjing Institute of Technology, Nanjing 211167, China; dingkq@njit.edu.cn (K.D.); yiwang@njit.edu.cn (Y.W.); lijuan_zhang@njit.edu.cn (L.Z.); why1871739065@163.com (H.W.); 2 Three Gorges Beijing Enterprises Nanjing Water Group Co., Ltd., Nanjing 210000, China; huangjing@bewg.net.cn; 3 School of Environmental Engineering, Xuzhou University of Technology, Xuzhou 221018, China; yangyy7075@126.com

**Keywords:** *Microcystis aeruginosa*, *Chlorella pyrenoidosa*, ultraviolet B radiation, photosynthetic efficiency, adaptation capability, nutrient enrichment

## Abstract

Frequent outbreaks of harmful algal blooms (HABs) represent one of the most serious outcomes of eutrophication, and light radiation plays a critical role in the succession of species. Therefore, a better understanding of the impact of light radiation is essential for mitigating HABs. In this study, *Chlorella pyrenoidosa* and non-toxic and toxic *Microcystis aeruginosa* were mono-cultured and co-cultured to explore algal responses under different nutrient regimes. Comparisons were made according to photosynthetically active radiation (PAR), UV-B radiation exerted oxidative stresses, and negative effects on the photosynthesis and growth of three species under normal growth conditions, and algal adaptive responses included extracellular polymeric substance (EPS) production, the regulation of superoxide dismutase (SOD) activity, photosynthetic pigments synthesis, etc. Three species had strain-specific responses to UV-B radiation and toxic *M. aeruginosa* was more tolerant and showed a higher adaptation capability to UV-B in the mono-cultures, including the lower sensitivity and better self-repair efficiency. In addition to stable μ_max_ in PAR ad UV-B treatments, higher EPS production and enhanced production of photosynthetic pigments under UV-B radiation, toxic *M. aeruginosa* showed a better recovery of its photosynthetic efficiency. Nutrient enrichment alleviated the negative effects of UV-B radiation on three species, and the growth of toxic *M. aeruginosa* was comparable between PAR and UV-B treatment. In the co-cultures with nutrient enrichment, *M. aeruginosa* gradually outcompeted *C. pyrenoidosa* in the PAR treatment and UV-B treatment enhanced the growth advantages of *M. aeruginosa*, when toxic *M. aeruginosa* showed a greater competitiveness. Overall, our study indicated the adaptation of typical algal species to ambient UV-B radiation and the stronger competitive ability of toxic *M. aeruginosa* in the UV-radiated waters with severer eutrophication.

## 1. Introduction

With the rapid economic development and pollutant discharge, eutrophication has seriously affected aquatic ecosystems over the last several decades [1,2]. Frequent outbreaks of harmful algal blooms (HABs) represent one of the most serious outcomes of eutrophication [3,4] and many studies have investigated the effects of environmental factors, such as temperature, light, and nutrients, on the growth of typical species and the development of HABs [5,6]. These factors could partly explain the underlying mechanism of HAB formation and the seasonal succession of species. For a long time, cyanobacteria gained much attention from environmental degradation and human health perspectives [7,8,9]. Especially, many scholars have focused on *Microcystis* in recent years, which is a dominant cyanobacterial genus in many eutrophic waters and often exhibits a greater threat to the microcystins produced by toxic species [10,11,12].

Light could directly affect the photosynthesis and growth of cyanobacteria [13,14], which were closely associated with light intensity, exposure time, and light wavelength. Especially, other than necessary photosynthetically active radiation (PAR; 400–700 nm), enhanced ultraviolet (UV) radiation is reported for many aquatic ecosystems throughout the world due to serious stratospheric ozone depletion [15,16]. Therefore, the effects of UV radiation on typical cyanobacterial species have received considerable attention in recent years, and most studies have used *Microcystis* as a model species [17,18,19]. Freshwater ecosystems in the middle and lower reaches of Yangtze River are susceptible to enhanced UV radiation due to the lack of depth refuge [20], and *Microcystis* often occur as the surface blooms that encounter higher irradiance [21,22]. It is assumed that *Microcystis* should be more threatened and suffered greater UV-induced damage. However, the frequency and intensity of the dominance of *Microcystis* continue to increase in typical eutrophic lakes in China, such as Lake Taihu. Hence, it is crucial to investigate and compare the responses of *Microcystis* and other algal species to UV radiation.

The composition of HABs in freshwater ecosystems is varied and often includes cyanobacteria and green microalgae as the major components [21,23]. For example, *Microcystis* and *Chlorella* were the most dominant species in eutrophic lakes in China, although their cell densities fluctuated wildly during different seasons [12]. Meanwhile, *Microcystis* blooms were often formed by mixed species when the seasonal succession and competition between the non-toxic and toxic species have been widely studied [24,25]. For example, the toxic *Microcystis aeruginosa* was determined to be more harmful to *Chlorella vulgaris* than the non-toxic species at higher temperatures [19]. Although numerous studies have focused on the effects of UV on algae in recent years, relatively few studies have deeply examined and compared the adaptive strategies to the ambient UV radiation of non-toxic and toxic *M. aeruginosa* and other species [16,26]. Some scholars investigated the effects of nutrient enrichment on algal responses to UV radiation and the results are varied. For example, Li et al. [27] reported that the effects of UV-B on phytoplankton productivity might be underestimated in iron-deficient ecosystems, and Yang et al. [28] reported that the negative impact was most pronounced when UV-B exposure and P limitation were combined. Meanwhile, Zheng et al. [29] reported that impacts of solar UV radiation on algal growth differed significantly at different N concentrations. However, the influence mechanisms of nutrient enrichment on algal adaptation and biotic interactions to UV radiation also remain unclear. In addition, many studies have investigated the effects of UV radiation on algal growth in the pure mono-culture systems [13,30], and it remains unclear how the coexistence of algae was affected by UV radiation, despite the fact that algal species coexist in the natural ecosystems. In this regard, the co-cultures with different nutrient conditions may provide useful information to address cyanobacterial blooms and algal competition in the natural waters and to better explain the synergistic effects of eutrophication and irradiation in mixed communities.

In this study, we selected *C. pyrenoidosa* and non-toxic and toxic *M. aeruginosa* to investigate their various physiological responses with ambient irradiation treatment under different nutrient regimes. The main goals were to: (i) analyze the effects and mechanisms of ambient UV-B radiation on three species, (ii) compare and explore the responses of the adaptation capability of three species to UV radiation, and (iii) study the effects of nutrient enrichment on algal growth and competition.

## 2. Materials and Methods

### 2.1. Algal Culture

*C. pyrenoidosa* (FACHB 5), non-toxic *M. aeruginosa* (FACHB 469), and toxic *M. aeruginosa* (FACHB 905) were obtained from the Freshwater Algae Culture Collection of the Institute of Hydrobiology, Chinese Academy of Sciences (FACHB). For the three algal species, *M. aeruginosa* is a dominant genus during the outbreaks of HABs, and *C. pyrenoidosa* was selected because of its common distribution and frequent co-existence with cyanobacteria in many Chinese eutrophic ecosystems [14]. All strains were pre-cultured separately and exponential growth was maintained by transferring 5 mL of growing cultures to fresh standard BG_11_ medium in Erlenmeyer flasks every 8–10 days for enlargement [31]. Pre-culture was performed under sterile conditions and the flasks were placed at 25 °C under 40 μmol photons m^−2^ s^−1^ PAR with cool white fluorescent lamps (light/dark regime of 12 h:12 h) in the illuminated incubator (GZX-250BS-II). All flasks were shaken three times per day to prevent the cells from adhering to inner walls, and the position of flasks was exchanged randomly to ensure uniform light exposure.

### 2.2. Experimental Setup

After pre-culture and enlargement–cultivation, the exponentially growing algal cells were collected and suspended in phosphate buffer solution (PBS, pH = 7.4) for washing and reservation prior to running our formal experiments. After 3–4 days, algal cells were collected again and inoculated into 500-mL flasks containing 300–400 mL of modified BG_11_ medium for experiments in the mono-cultures and co-cultures. In the first scheme of modified BG_11_ medium, the composition was as shown in Table S1, and concentrations of nitrogen (N), phosphorus (P), and iron were comparable to those in the natural waters, representing normal growth conditions. In the second scheme of modified BG_11_ medium, the initial N, P, and iron concentrations were appropriately increased (Table S1), representing nutrient enrichment conditions. The initial cell density of three species was 1.0 × 10^6^ cells mL^−1^, which approximated the cell number at the beginning of HABs in most eutrophic lakes in China [32]. Meanwhile, co-cultures were conducted to simulate natural conditions and to explore the characteristics of algal competition. To this end, *C. pyrenoidosa* was co-cultured with non-toxic and toxic *M. aeruginosa*, when the inoculation ratio was 1:1 and the cell density of each strain was 1.0 × 10^6^ cells mL^−1^.

On each day, algal cultures in the flasks were transferred into sterilized petri dishes with quartz glass covers (20 cm in diameter) for PAR or UV-B exposure after slightly shaking the flasks, representing PAR treatment and UV-B treatment, and the two treatments both lasted for 4 h (9:00–13:00). In the PAR treatment, petri dishes were maintained in another illumination incubator and continuously irradiated with 50 μmol photons m^−2^ s^−1^ PAR. In the UV-B treatment, petri dishes were stored in clean chambers and subjected to high-pressure mercury UV-B lamps with the dominant wavelength of 313 nm (TL20W/01RS, Philips, Eindhoven, Netherlands, Figure S1). In the UV-B treatment, light exposure was restricted to UV-B and no photosynthetically active wavelengths were given to algal cells. The effective irradiation intensity of PAR and UV-B in our study was 70 and 0.8 W m^−^^2^, respectively. After the irradiation treatment for 4 h, algal cultures were returned to flasks and incubated under the conditions as described in the pre-cultures for the rest time on each day (dark during 0:00–6:00 and 18:00–24:00, 40 μmol photons m^−2^ s^−1^ PAR during 6:00–9:00 and 13:00–18:00). The incubation lasted for 14 days in our study and a schematic diagram of the experiment is shown in Figure S2.

Based on field monitoring, the adopted PAR and UV-B in different treatments was in accordance with the natural conditions at noon in the middle and lower reaches of the Yangtze River [26]. The vertical sides of petri dishes were all covered with aluminum foil to ensure vertical radiation and the irradiance was measured using a miniature fiber optic spectrometer (FLA4000A+, Flight, Hangzhou, China).

### 2.3. Analytical Methods of Parameters

#### 2.3.1. Cell Density and Photosynthetic Efficiency

Subsamples were regularly taken for determining cell density in the mono-cultures and co-cultures. Cells were both enumerated by using a flow cytometer (CytoFLEX S, Beckman Coulter, Fullerton, CA, USA), when *M. aeruginosa* and *C. pyrenoidosa* could be clearly differentiated by autofluorescence in the co-cultures [33]. Then, the algal growth rate was determined as follows: μ = (ln*N*_2_ − ln*N*_1_)/(t_2_ − t_1_), where *N*_1_ and *N*_2_ was the cell density on days t_1_ and t_2_, respectively. The maximum μ during the whole incubation period was defined as μ_max_, which is an important index to indicate algal growth potential. 

A Phyto-PAM fluorometer (Hein Walz, Effeltrich, Germany) was adopted to determine the effective quantum yield (*F_v_*/*F_m_*) of algal species. The Phyto-PAM fluorometer has been increasingly used in laboratory and in situ experiments, and *F_v_*/*F_m_* can effectively indicate the efficiency of algal photosynthesis apparatus [34,35,36].

#### 2.3.2. Release of K^+^ by Algal Cells

As K^+^ is absorbed into the vacuole of algae cells and is mainly stored as an enzyme activator, the algal release of K^+^ can be manifested for cell membrane damage [37,38]. During the incubation process, 10-mL supernatant samples of three species were regularly taken and immediately filtered through 0.2-μm mixed cellulose ester filters (Whatman, Little Chalfont, Buckinghamshire, UK) after daily irradiation. Then, the solution was acidified to pH = 2 with HNO_3_ and K^+^ content was determined by the inductively coupled plasma mass spectrometry (IC-PMS) (XII series, Thermo, Waltham, MA, USA). Afterwards, the release rate of K^+^ by algal cells was calculated as a percentage and ultrasonic disrupted samples were adopted to make a comparison.

#### 2.3.3. Characterization of Extracellular Polymeric Substance (EPS)

The extraction of EPS was conducted according to the methods described by Gao et al. [39] and Yang et al. [40]. Firstly, samples of the algal cultures were sonicated with 100 W ultrasound treatment for 5 min to obtain a uniform distribution, then filtered through 0.45-μm filters (Whatman, Little Chalfont, Buckinghamshire, UK) in order to separate soluble EPS (SEPS) from cell pellets [41]. The supernatant was collected and stored at 4 °C in the dark. Then, the harvested cells were washed in ultra-pure water, re-suspended in 0.05% NaCl solution, and centrifuged at 16,000× *g* for 20 min. The resulting supernatant was collected as bounded EPS (BEPS) and also stored at 4 °C in the dark.

On Day 1, the filtered SEPS and BEPS fractions were taken without dilution and the excitation emission matrix (EEM) spectra were determined by using a fluorescence spectrometer (F-7000, Hitachi, Tokyo, Japan). The excitation wavelengths were increased from 200 to 400 nm in 5-nm steps and the emission spectra were recorded from 250 to 500 nm in 1-nm increments. The increments were all set at 5 nm, and a scan speed of 2400 nm min^−1^ was applied. The blank scans were performed using modified BG_11_ medium, in which no fluorescence substance was present.

In addition, the contents of SEPS and BEPS were quantified spectrophotometrically (UV-2700, Shimadzu, Kyoto, Japan) during the whole incubation process by the anthrone sulfuric acid method and the values were normalized to cell density [42].

#### 2.3.4. Reactive Oxygen Species (ROS) in Cells and Superoxide Dismutase (SOD) Activity

Before and during the incubation process in the mono-cultures, subsamples were regularly taken for the determination of ROS in algal cells of three species and activity of SOD. The details can be seen in the Supplementary Materials (SM).

#### 2.3.5. Cell Adsorption Spectra and Contents of Photosynthetic Pigments

At the beginning of incubation, a scanning spectrophotometer (Beckman Coulter, Fullerton, CA, USA) was used to measure the whole-cell absorption spectra of three species between 400 and 750 nm. Cell cultures with the optical density value at 680 nm (OD_680_) of 0.10 were used for measurement and the absorption peaks could indicate the existence of photosynthetic pigments in algal cells [43].

At different stages of the incubation (Day 1 and 8) in the mono-cultures, a subsample of algal cultures was collected and filtrated through 0.2-mm mixed cellulose ester filters (Whatman) to determine the contents of photosynthetic pigments (pg cell^−1^) in single cells, including chlorophyll a (Chl-a), carotenoid (CAR), and phycocyanin (PC). The details can be seen in SM.

### 2.4. Statistical Analysis

All experiments were conducted in triplicate and means ± standard deviations of three replicates were calculated. The parametric three-way repeated-measures analysis of variance (RM-ANOVA) was used to determine the effects of irradiation treatments (PAR and UV-B), species (*C. pyrenoidosa*, non-toxic and toxic *M. aeruginosa*), and sampling time on the cell density, growth rates, EPS contents, and other parameters. Data were tested for normality and the variance assumptions of parametric ANOVA, and no data transformation was needed. If the interaction factor was significant at *p* < 0.05, a one-way ANOVA followed by Tukey’s test was adopted to determine where differences lie. Meanwhile, the student’s *t*-test was adopted to test the differences in the algal cell density of different species on a specific day in the co-cultures. All statistical analyses were performed using SPSS 22.0 (Chicago, IL, USA).

## 3. Results

### 3.1. Algal Growth in the Mono-Cultures under Normal Growth Conditions

#### 3.1.1. Cell Density and Algal Photosynthetic Efficiency

**Figure 1:**
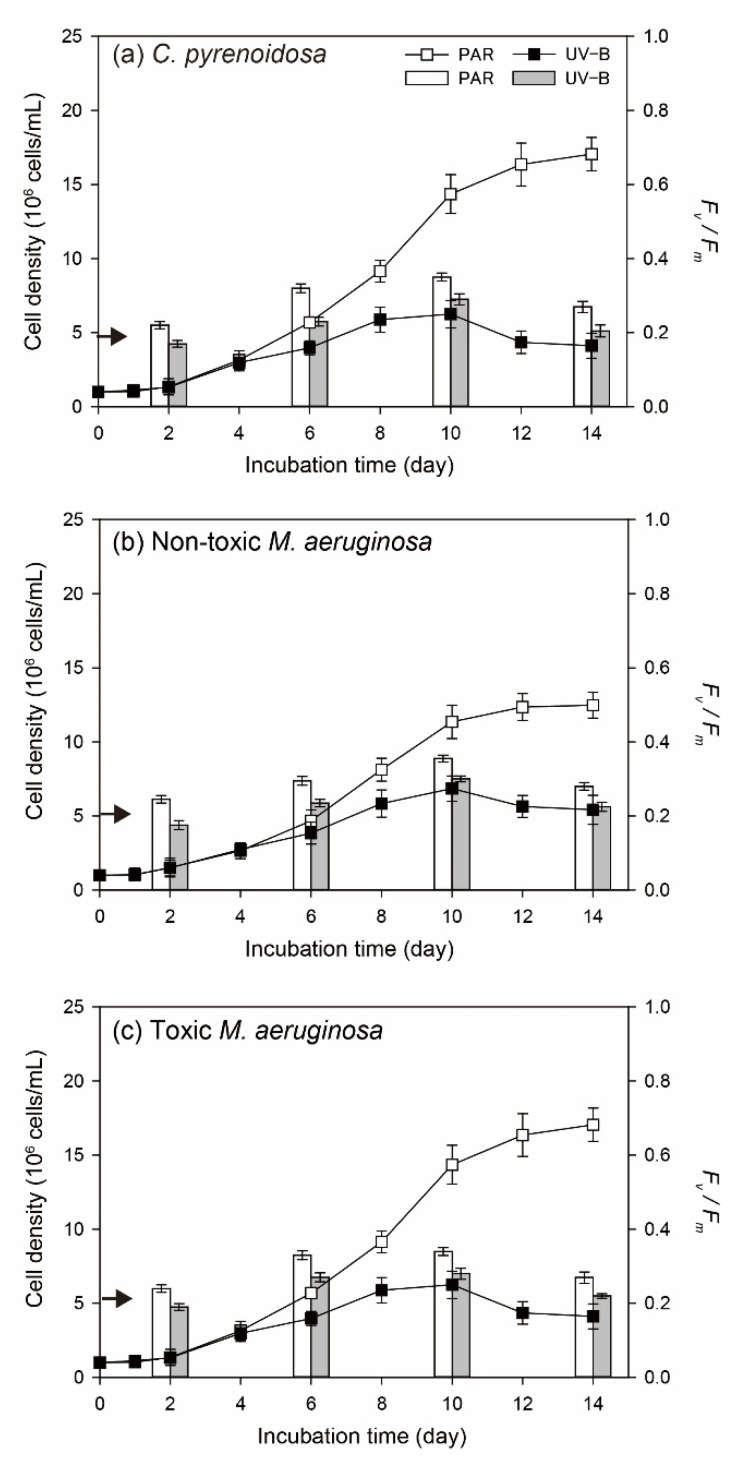
Cell density (line and scatter) and *F_v_/F_m_* (vertical bar) of three species in the PAR and UV-B treatments under normal growth conditions (the arrow indicates the initial value of *F_v_/F_m_*).

In the PAR treatment, three species could all persistently grow and reached the maximum cell density on Day 14 (Figure 1). Meanwhile, the maximum cell density of *C. pyrenoidosa* was higher compared to the other two species, and toxic *M. aeruginosa* propagated more slowly. By comparison, the significant inhibitive effects (*p* < 0.05) of UV-B radiation were observed on algal growth, including cell density on a specific day, the maximum cell density during the incubation, and the duration of exponential growth. More specifically, three species both grow slowly and their cell densities began to decrease on Day-10 in the UV-B treatment. For algal photosynthetic efficiency, *F_v_/F_m_* of three species in the PAR treatment gradually increased before Day-10 and decreased afterwards. The variation patterns of algal *F_v_/F_m_* were different in the UV-B treatment, which decreased from the beginning, increased during Day 2–10, and declined afterwards. Moreover, algal *F_v_/F_m_* on a specific day in the UV-B treatment was always lower (*p* < 0.05) than that in the PAR treatment.

**Figure 2:**
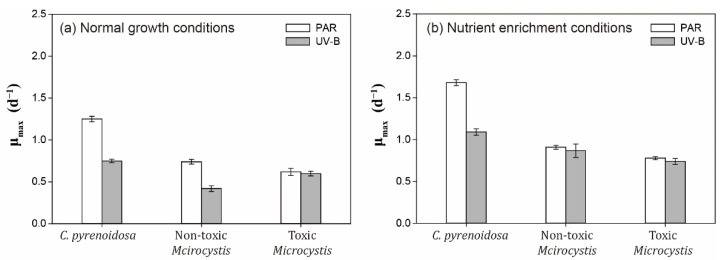
Maximum growth rates of three species in the PAR and UV-B treatment under normal growth conditions and nutrient enrichment conditions.

For *C. pyrenoidosa* and non-toxic *M. aeruginosa* under normal growth conditions, μ_max_ in the UV-B treatment was significantly lower (*p* < 0.05) compared with those in the PAR treatment (Figure 2), which indicated the negative effects of UV-B on the intrinsic growth potential of two species. Although μ_max_ of toxic *M. aeruginosa* was lower in the PAR treatment compared with the other two species, it did not change significantly (*p* > 0.05) with daily UV-B radiation in our study.

#### 3.1.2. Diurnal Changes of Algal *F_v_/F_m_*

**Figure 3:**
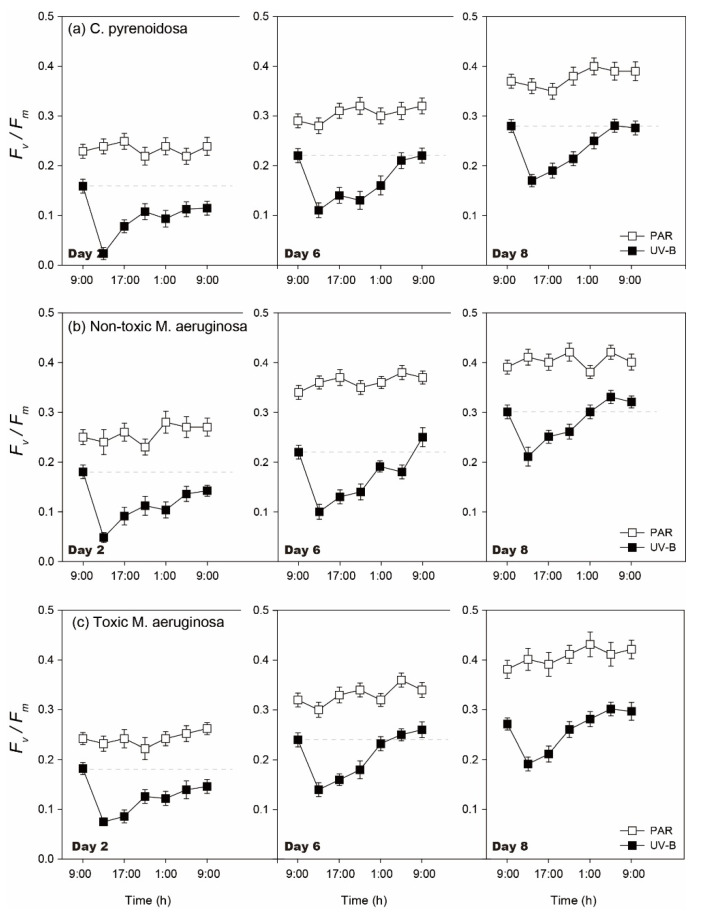
Diurnal changes of *F_v_/F_m_* of three species in the PAR and UV-B treatments under normal growth conditions on Day 2, Day 6, and Day 8.

The diurnal changes of *F_v_/F_m_* were similar for three species in the PAR and UV-B treatments (Figure 3). During the incubation, diurnal *F_v_/F_m_* did not change significantly after 4 h of PAR treatment (*p* > 0.05), irrespective of species. In comparison, algal diurnal *F_v_/F_m_* decreased after 4 h of UV-B radiation (*p* < 0.05), which could then increase after the withdrawal of UV-B radiation on each day. However, the decline degree and recovery of *F_v_/F_m_* was dependent on the incubation time and species.

For three species, their *F_v_/F_m_* decreased to 15.2–40.6% of the initial values after UV-B radiation on Day 2 and they recovered to 75.2–83.3% of the initial values within 20 h, when the decline was lower for toxic *M. aeruginosa* (*p* < 0.05). On Days 6 and 8, the decline of algal *F_v_/F_m_* worsened after UV-B radiation and algal *F_v_/F_m_* could all recover to the initial values within 20 h. Meanwhile, the recovery rate increased with the development of incubation, and the recovery rate was highest for toxic *M. aeruginosa* (*p* < 0.05). In comparison, the inhibition of UV-B radiation on *F_v_/F_m_* was maximum for *C. pyrenoidosa* on each day and the recovery rate of its *F_v_/F_m_* was lower.

### 3.2. K^+^ Contents in the Algal Cultures

**Figure 4:**
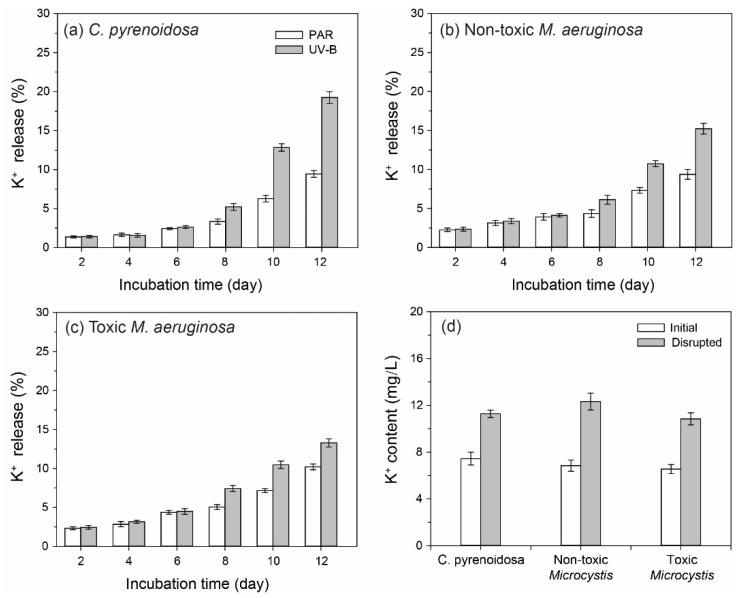
(**a**–**c**) The release rates of K^+^ by three species in the PAR and UV-B treatments in the mono-cultures. (**d**) A comparison of the initial and ultrasonic disrupted cells of three species.

The release rates of K^+^ by three species were all less than 5% before Day 6 in the PAR treatment (Figure 4), which could indicate the integrity of cells. Moreover, algal release rates of K^+^ did not differ significantly between PAR and UV-B treatments during this period (*p* > 0.05), when the death and propagation of algal cells might be in a state of balance. After reaching the exponential growth stage in the PAR treatment, algal metabolism was enhanced with higher cell density, leading to the gradual increase of K^+^ in the cultures (9.38–10.22% on Day 12). In comparison, the cell rupture of three species and algal release rates of K^+^ were significantly promoted in the UV-B treatment (*p* < 0.05), indicating the greater damage of UV-B radiation on algal cells during this period.

### 3.3. EPS Determination of Algal Cells

At the beginning of incubation, 3-D EEM spectra of algal EPS were determined and EEM contours were depicted (Figure S3). Results showed that EEM contours of BEPS and SEPS were similar for *C. pyrenoidosa* and non-toxic and toxic *M. aeruginosa*, indicating the similar metabolism patterns of three species. For BEPS, two peaks were presented near Ex/Em of 225/325 nm (peak T_2_) and 280/325 nm (peak T_1_), which belonged to the low-molecular aromatic protein and soluble microbial by-product like protein (such as tyrosine and tryptophan-like substances), respectively [44]. In contrast, three peaks were presented near Ex/Em of 280/325 nm (peak T_1_), 340/430 nm (peak C, humic-acid like substances), and 275/435 nm (peak A, fulvic-acid like substances) in SEPS.

**Figure 5:**
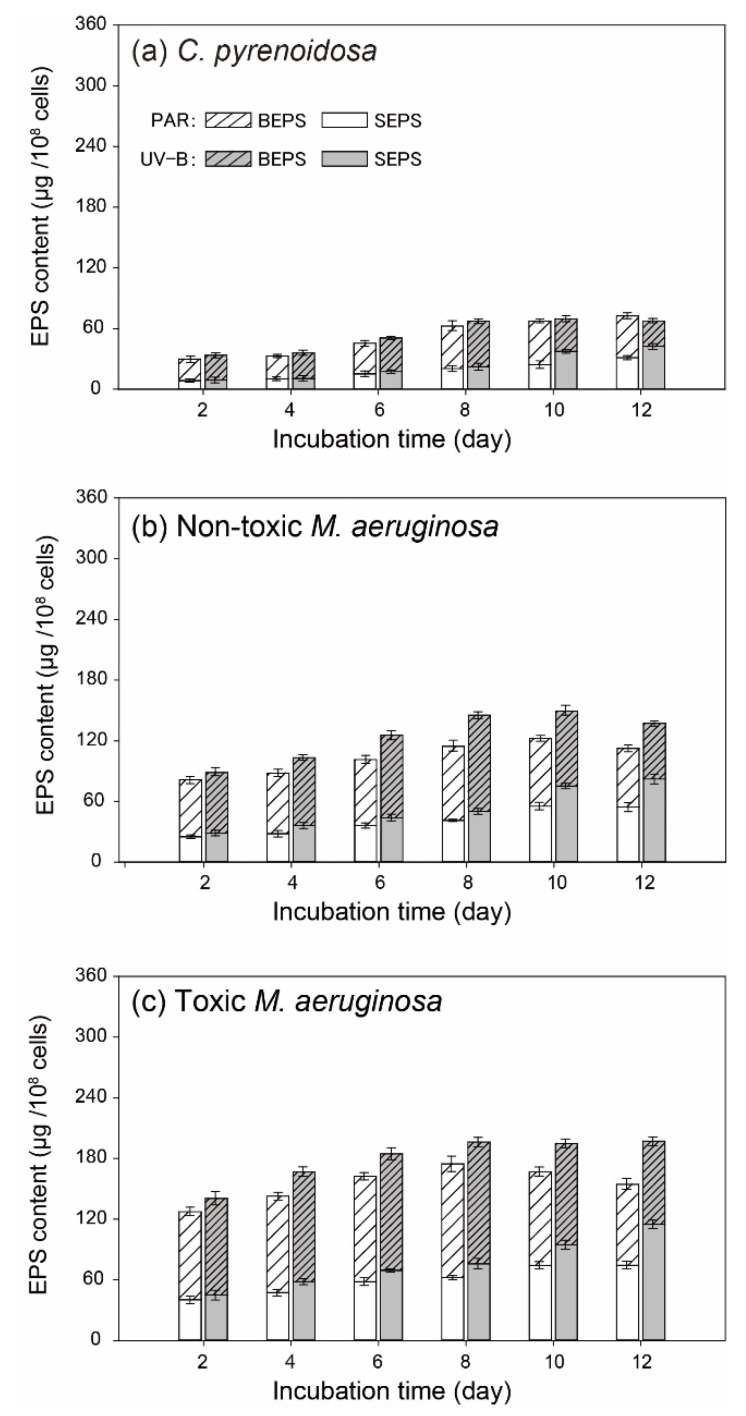
Contents of BEPS and SEPS produced by (**a**) *C. pyrenoidosa*, (**b**) non-toxic *M. aeruginosa* and (**c**) toxic *M. aeruginosa* cells in the PAR and UV-B treatments.

During the incubation, EPS contents increased for all three species in the PAR and UV-B treatments, and SEPS and BEPS had distinct changing trends during the incubation (Figure 5). For two *M. aeruginosa* species in the PAR treatment, SEPS content gradually increased and then remained constant, but BEPS content both increased in the early stage and decreased with increasing cell density. Meanwhile, the production of BEPS and SEPS was stronger for toxic *M. aeruginosa* (*p* < 0.05) during the incubation. In comparison, algal production of BEPS and SEPS by *M. aeruginosa* species was enhanced (*p* < 0.05) before Day 8 in the UV-B treatment. With the decline of cell densities, BEPS contents of two *M. aeruginosa* species decreased and their SEPS contents increased greatly after Day 10. In contrast, EPS production by *C. pyrenoidosa* was weaker in the PAR treatment (*p* < 0.05) and UV-B radiation did not significantly promote BEPS production before Day 8.

### 3.4. Antioxidant Responses of Algal Species under Normal Growth Conditions

#### 3.4.1. ROS in Algal Cells and SOD Activity

**Figure 6:**
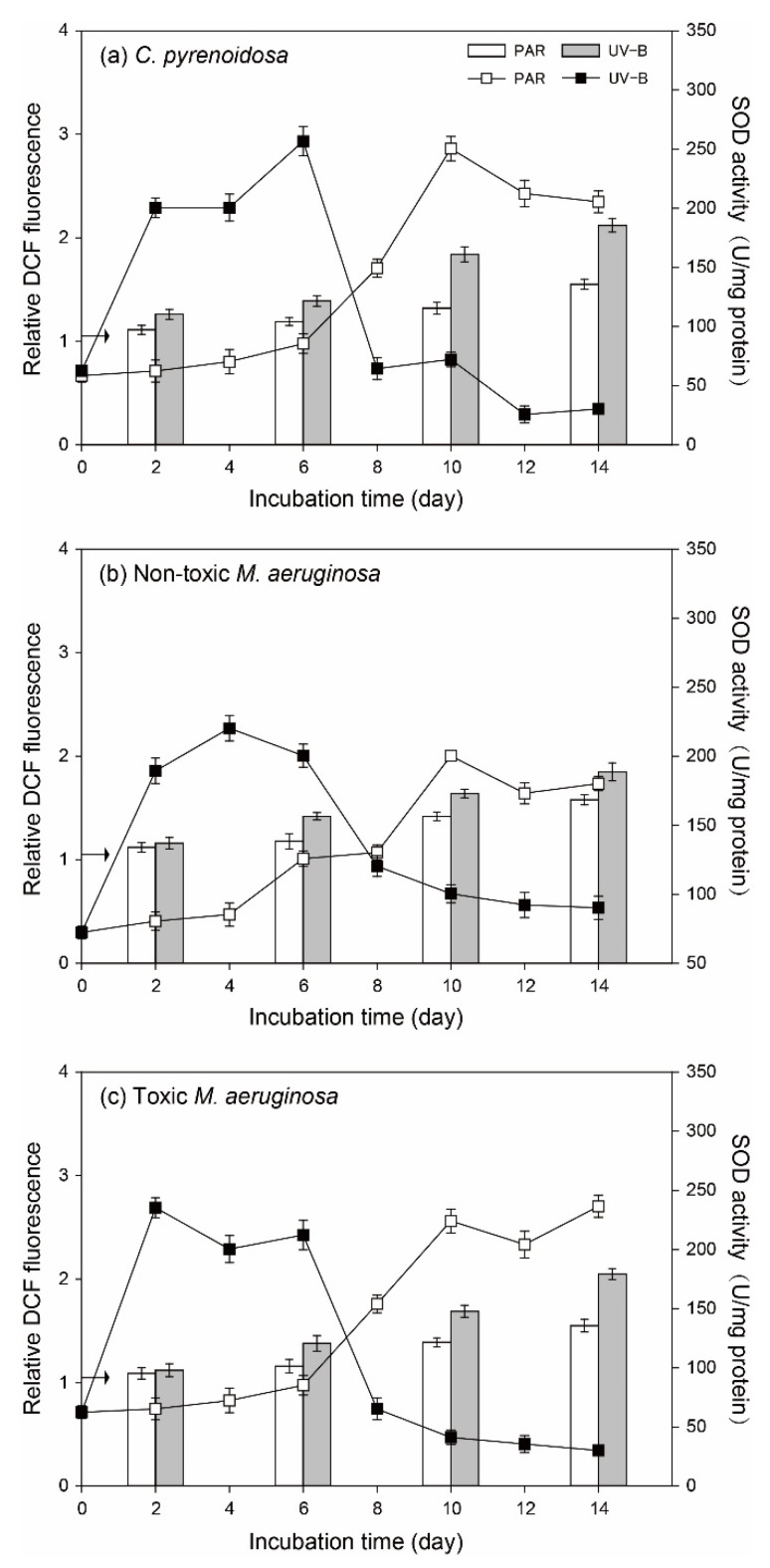
ROS in the cells of three species (vertical bar) and algal SOD activity (line and scatter) during the incubation in the PAR and UV-B treatments under normal growth conditions (the arrow indicates the initial value of ROS contents).

The variation patterns of ROS in algal cells and algal SOD activity were similar for three species (Figure 6). In the PAR treatment, ROS and algal SOD activities were constant (*p* > 0.05) before Day 4 compared to the initial values, indicating that PAR treatment did not cause evident oxidative stresses on three species. However, ROS gradually increased after Day 6 in the PAR treatment, and algal SOD activity was elevated for all three species. Compared with PAR treatment, ROS of two *M. aeruginosa* species were comparable in the UV-B treatment on Day 2 and they were higher (*p* < 0.05) after Day 6. In comparison, ROS of *C. pyrenoidosa* was higher in the UV-B treatment on Day 2 and it was further promoted after Day 6, which was higher than that in the cells of two *M. aeruginosa* species. Irrespective of species, algal SOD activities were significantly higher (*p* < 0.05) in the UV-B treatment before Day 8, which could provide effective antioxidant protection. However, as the incubation progressed, algal SOD activity decreased sharply and leveled off until the end of incubation.

#### 3.4.2. Contents of Photosynthetic Pigments

For all three species, the whole-cell absorption spectra indicated that they have Chl-a (two absorption peaks in the blue and red parts of the spectra at around 440 and 680 nm) and CAR with an absorption peak at around 495 nm (Figure S4). Moreover, non-toxic and toxic *M. aeruginosa* had an extra absorption peak at around 620 nm, which was regarded to phycocyanin in cyanobacterial cells [45].

Similar Chl-a contents of algal single cells were observed in the PAR and UV-B treatments on Day 1 (*p* > 0.05, Table 1). However, CAR of three species and PC of two *Microcystis* species were significantly promoted (*p* < 0.05) in the UV-B treatment on Day 1. Moreover, algal CAR/Chl-a and PC/Chl-a ratios in the UV-B treatment were higher (*p* < 0.05) than those in the PAR treatment at this moment. As incubation progressed on Day 8, Chl-a in algal cells were lower (*p* < 0.05) in the UV-B treatment compared to those in the PAR treatment, which could indicate the damage to chlorophyll synthesis. Meanwhile, algal CAR and PC contents in the UV-B treatment decreased greatly (*p* < 0.05) compared with Day 1, which were lower (*p* < 0.05) than those in the PAR treatment. Compared with PAR treatment, the CAR/Chl-a ratio of *C. pyrenoidosa* on Day 8 was lower in the UV-B treatment (*p* < 0.05), whereas CAR/Chl-a and PC/Chl-a ratios of two *M. aeruginosa* species were significantly higher on Day 8 in the UV-B treatment (*p* < 0.05).

### 3.5. Algal Growth in the Mono-Cultures under Nutrient Enrichment Conditions

#### 3.5.1. Cell Density and Algal Photosynthetic Efficiency

**Figure 7:**
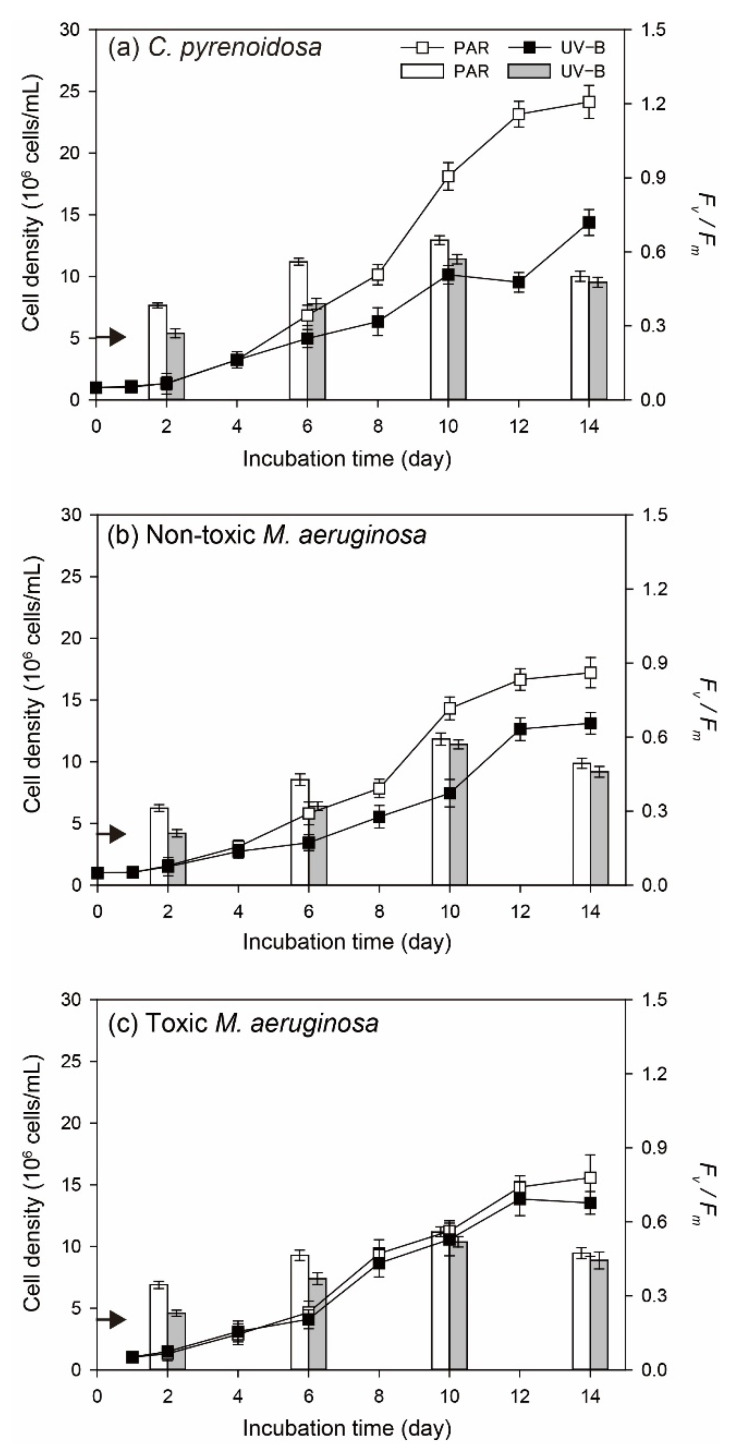
Cell density (line and scatter) and *F_v_/F_m_* (vertical bar) of three species in the PAR and UV-B treatments under nutrient enrichment conditions (the arrow indicates the initial value of *F_v_/F_m_*).

Compared to normal growth conditions, three species all grew steadily under nutrient enrichment conditions and their cell densities did not decrease at the later stage (Figure 7). For *C. pyrenoidosa* and non-toxic *M. aeruginosa*, their cell densities on a specific day and the maximum cell density in the UV-B treatment were significantly lower (*p* < 0.05) than those in the PAR treatment. However, no significant difference in the cell density of toxic *M. aeruginosa* (*p* > 0.05) was observed between PAR and UV-B treatments during the whole incubation process. *F_v_/F_m_* of three species gradually increased and decreased afterwards in both PAR and UV-B treatments. Although algal *F_v_/F_m_* in the UV-B treatment were lower (*p* < 0.05) than that in the PAR treatment before Day 6, the difference became smaller at the later stage of incubation. For example, *F_v_/F_m_* of non-toxic and toxic *M. aeruginosa* were both comparable (*p* > 0.05) in the UV-B and PAR treatments on Day 10 and Day 14.

As expected, μ_max_ of three species increased with nutrient enrichment compared with those under normal growth conditions in the PAR and UV-B treatments (Figure 2). μ_max_ of non-toxic and toxic- *M. aeruginosa* were lower compared with *C. pyrenoidosa*, but they were both comparable between PAR and UV-B treatments (*p* > 0.05). 

#### 3.5.2. Diurnal Changes of Algal *F_v_/F_m_*

**Figure 8:**
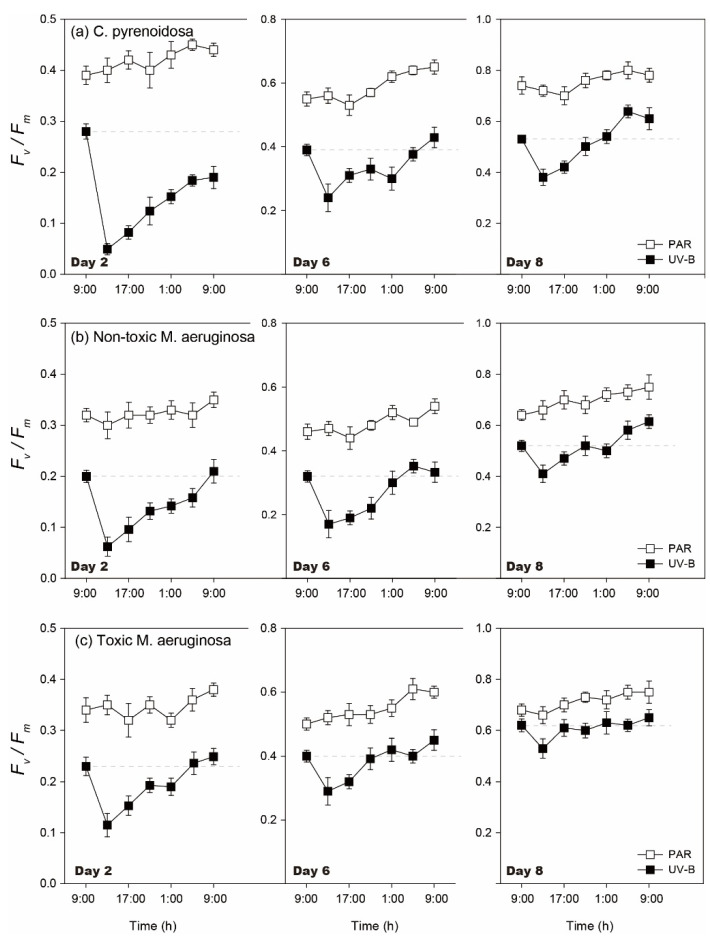
Diurnal changes of *F_v_/F_m_* of three species in the PAR and UV-B treatments under nutrient enrichment conditions on Day 2, Day 6, and Day 8.

Compared with normal growth conditions, diurnal changes of algal *F_v_/F_m_* were similar under nutrient enrichment conditions (Figure 8). However, the decline degrees of algal *F_v_/F_m_* after UV-B radiation were lower (17.5–50.8%), and the recovery efficiency of *F_v_/F_m_* was better with nutrient enrichment. For example, *F_v_/F_m_* of two *M. aeruginosa* species could both totally recover to the initial values after UV-B radiation on Day 2, and *F_v_/F_m_* of *C. pyrenoidosa* on Day 6 and Day 8 totally recovered to the initial values within 16 h and 8 h after UV-B radiation, respectively. For three species in the UV-B treatment, the decline degree of *F_v_/F_m_* was also lower for toxic *M. aeruginosa* and it exhibited a faster recovery rate. This result was consistent with that under normal growth conditions.

### 3.6. Antioxidant Responses of Algal Species under Nutrient Enrichment Conditions

#### 3.6.1. ROS in Algal Cells and SOD Activity

**Figure 9:**
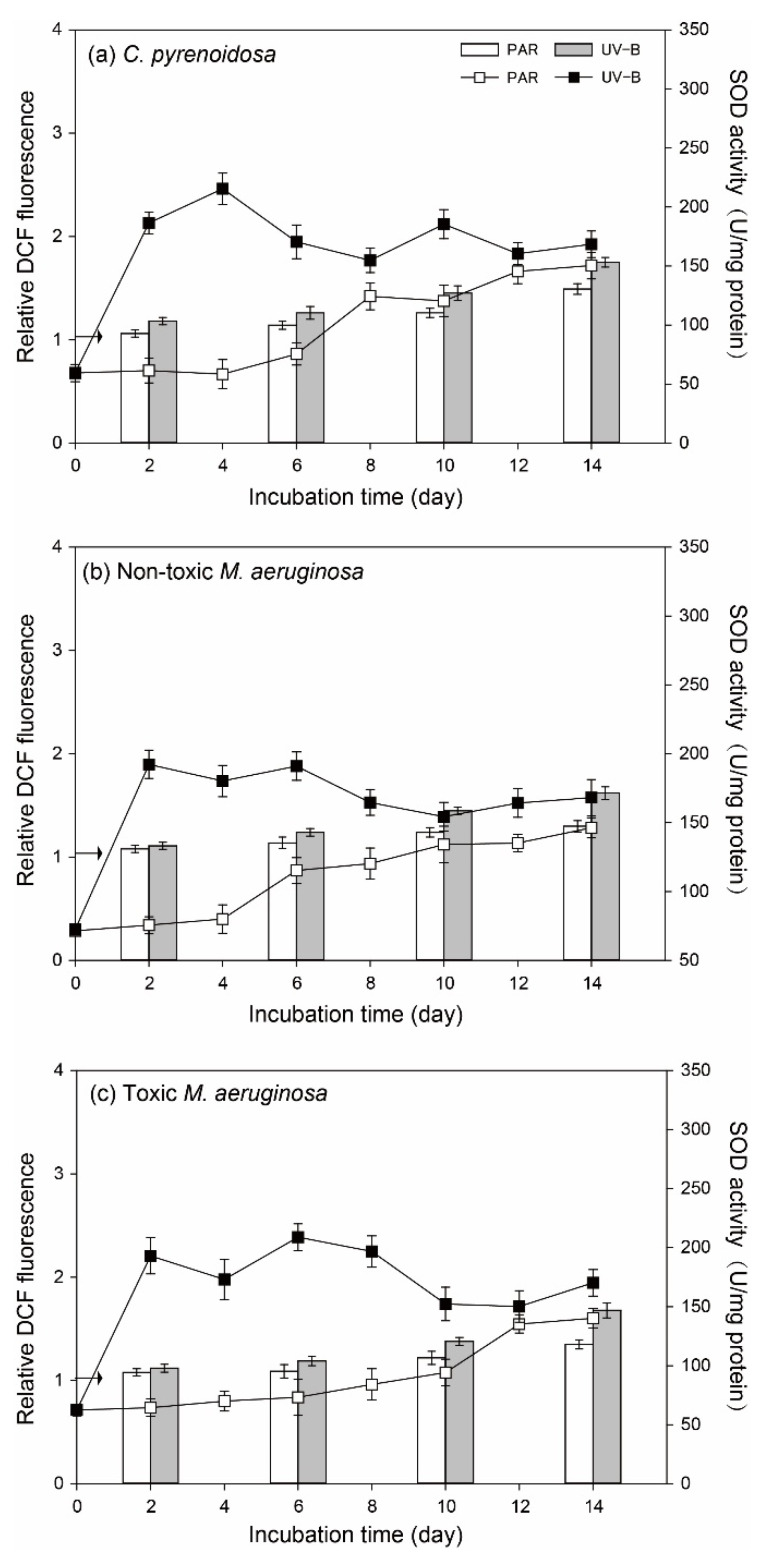
ROS in the cells of three species (vertical bar) and algal SOD activity (line and scatter) during the incubation in the PAR and UV-B treatments under nutrient enrichment conditions (the arrow indicates the initial value of ROS contents).

Under nutrient enrichment conditions, the variation patters of ROS in algal cells and algal SOD activity were also similar for three species (Figure 9). More specifically, PAR treatment did not cause great oxidative stresses on algae, but ROS and algal SOD activity gradually increased at the later stage of incubation. In the UV-B treatment, ROS in algal cells also increased gradually, and they only showed higher values (*p* < 0.05) than those in the PAR treatment after Day 10. For algal SOD activity in the UV-B treatment, they all exhibited a sharp increase and decreased gradually to maintain a stable value. For both PAR and UV-B treatments, ROS in algal cells were lower (*p* < 0.05) than those under normal growth conditions on a specific day.

#### 3.6.2. Contents of Photosynthetic Pigments

As shown in Table 2, similar patterns were overserved for the algal synthesis of photosynthetic pigments on Day 1. Compared to the initial values, Chl-a contents of algal single cells were comparable in the PAR and UV-B treatment (*p* > 0.05), but CAR and PC in single cells increased greatly (*p* < 0.05) in the UV-B treatment, resulting in the higher CAR/Chl-a and PC/Chl-a ratios of three species on Day 1. Moreover, CAR and PC contents, CAR/Chl-a and PC/Chl-a ratios were all higher (*p* < 0.05) with nutrient enrichment compared to those under normal growth conditions.

On Day 8, despite the fact that the Chl-a contents of algal single cells were lower (*p* < 0.05) in the UV-B treatments, they showed an increasing trend compared with those on Day 1. This was consistent with the patterns of cell density. In addition, CAR and PC in single cells were also higher (*p* < 0.05) in the UV-B treatment at this moment, and CAR/Chl-a and PC/Chl-a ratios were promoted with UV-B radiation. This pattern was remarkably different from that under normal growth conditions.

### 3.7. Interspecific Competition in the Co-Cultures

**Figure 10:**
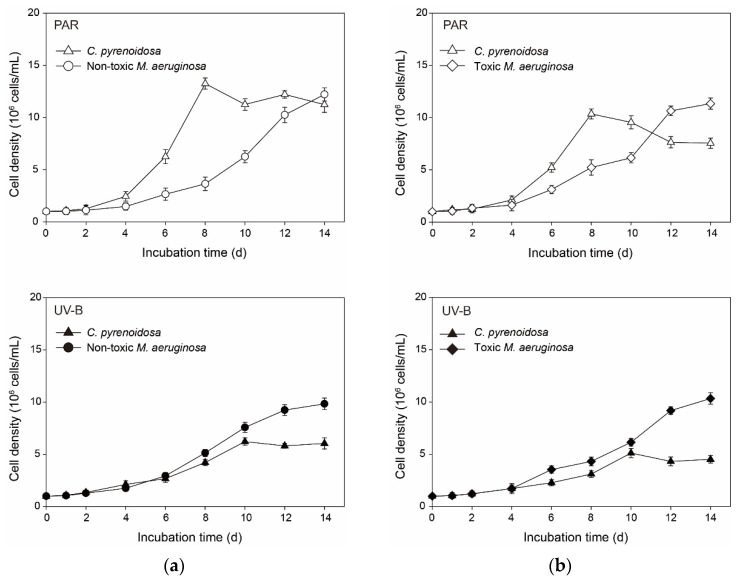
Cell densities of algal species in the co-cultures of (**a**) *C. pyrenoidosa* & non-toxic *M. aeruginosa*, and of (**b**) *C. pyrenoidosa* & toxic *M. aeruginosa* in the PAR and UV-B treatments under nutrient enrichment conditions.

Algal growth patterns were comparable in the PAR treatment (Figure 10), i.e., *C. pyrenoidosa* grew rapidly after the lag period and soon outcompeted non-toxic or toxic *M. aeruginosa*, while the cell density of *C. pyrenoidosa* decreased when that of *M. aeruginosa* started to increase. Compared with mono-cultures, the maximum cell densities of three species were all lower (*p* < 0.05) in the co-cultures (Table S2). However, the maximum cell density of *C. pyrenoidosa* seemed to decrease more in the PAR treatment, and the decline was great in the co-cultures of *C. pyrenoidosa* with toxic *M. aeruginosa*. In comparison, two *M. aeruginosa* species gained the obvious dominance and maintained competitive advantages from the beginning in the UV-B treatment, and the growth of *C. pyrenoidosa* was also markedly inhibited, which achieved 42.1% and 31.4% of the maximum cell density in the mono-cultures. Meanwhile, despite the faster growth of *C. pyrenoidosa* in the mono-cultures, μ_max_ of *C. pyrenoidosa* decreased greatly when it was co-cultured with *M. aeruginosa* in the PAR and UV-B treatments. However, μ_max_ of non-toxic *M. aeruginosa* only decreased slightly and μ_max_ of toxic *M. aeruginosa* even increased slightly.

## 4. Discussion

### 4.1. Effects of UV-B Radiation and Algal Responses

Although UV irradiance usually constitutes a few percent of solar radiation (5.85–8.51% in China), many studies have analyzed the effects of UV radiation on algal growth and negative effects were often reported [46,47]. Based on field and laboratory experiments, the main influencing mechanisms of UV-B radiation on algae include cell vitality impairment, ROS production, DNA damages, changes of nutrient utilization, etc. [26,27,48,49]. Our results are consistent with these findings, namely in that ambient UV-B radiation could exert negative effects on typical algal species in freshwater ecosystems, which also showed adaptative responses to UV-B radiation.

Since the growth and vitality of photosynthetic organisms are mainly governed by photosynthetic activity and the photosynthetic apparatus is an important damage target of UV-B radiation [50,51], daily *F_v_/F_m_* of all three species in the UV-B treatment were often lower compared to those in the PAR treatment. This result indicated that ambient UV-B might cause damages to D1 or D2 protein in algal photosystems [52] and 50 μmol m^−2^ s^−1^ of PAR did not have similar effects. However, different from many other studies using high-dose UV-B radiation whereby algal photosynthetic systems were greatly damaged [53,54], algal *F_v_/F_m_* gradually increased during Day 2–10 under two different growth conditions. Based on algal Chl-a contents on Day 1 and the release rates of K^+^, the adopted UV-B treatment in our study did not have direct lethal effects on algae, and changes of algal *F_v_/F_m_* and growth could be the balance between the light-induced effects and adaptive physiological processes of cells. This was confirmed by the diurnal changes of algal *F_v_/F_m_*, as *F_v_/F_m_* of three species recovered with different rates after UV-B exposure, which could have resulted from processes, such as oxidation resistance, nucleotide resynthesis, ATP supply, or the repair of damaged proteins [55,56].

UV radiation could cause the overexcitation of substances and produce excess ROS in algal cells or in the cultures [57,58], leading to the impairment of algal photosynthetic systems and normal growth. Consistently, ROS contents in UV-B radiated algal cells were higher during the incubation under normal growth conditions. The increase of ROS in the later period in the PAR treatment was consistent with the work of Latifi et al. [59] in that some environmental factors, such as nutrient deficiency and light limitation, could indirectly generate ROS at multiple sites of the photosynthetic electron transport chain in algal cells. However, as mentioned above, the oxidative stresses and resulting damages could be mitigated with algal adaptive strategies. In our study, EPS production, the up-regulation of SOD activity, CAR and PC synthesis, and recovery of *F_v_/F_m_* by three species, could act as their effective adaptation mechanisms, resulting in decreased sensitivity to UV-B exposure and increased self-repair efficiency. For example, algal EPS consisted of polysaccharides, proteins, lipids, and humic substances and often appeared as a structureless slimy layer around cells, which was helpful to algal aggregation and its resistance to environmental stresses [38,42,60]. Meanwhile, higher CAR and PC in cells could adsorb UV-B light and quench ROS to alleviate damage to algal photosynthetic systems and DNA [50,61]. Moreover, higher CAR in the cells could increase the algal utilization efficiency of light and promote its generation of ATP and other substances [50,62], such as antioxidant enzymes, nucleotides, and proteins, to repair damaged apparatus in algal cells [18,30]. In the UV-B treatment, the high SOD activity and enhanced production of CAR and PC by algae in the early stage could partly explain the gradual increase of algal *F_v_/F_m_* and cell density. However, these adaptive responses of three species might be not enough to remove UV-induced oxidative stresses at the later stage under normal growth conditions, and inhibition on algal growth could occur. Our previous study indicated that algal adaptation to UV-B radiation required energy and essential nutrient substances [26], and this could be the possible reason for algal decay at the later stage in the UV-B treatment. Especially, cumulative ROS might damage the antioxidant systems of UV-radiated algae after Day 8 under normal growth conditions and result in low SOD activity and algal death.

### 4.2. Comparison of Algal Adaptation to UV-B Radiation

In previous studies, scholars have often investigated the strategies of cyanobacteria to alleviate the harmful effects of UV-B radiation, such as the production of UV-absorbing compounds (UVCs) to mitigate photo-induced damages, vertical migration of cells to decrease the irradiation stress, enhanced self-repair, etc. [46,51,63]. In this study, three species exhibited strain-specific responses to UV-B radiation, when toxic *M. aeruginosa* was more tolerant and showed a higher adaptation capability, including lower sensitivity to UV-B radiation and better self-repair efficiency.

Firstly, *C. pyrenoidosa* grew faster, whereas toxic *M. aeruginosa* had similar μ_max_ in the PAR and UV-B treatments, which might indicate the stronger plasticity of toxic *M. aeruginosa* to maintain a stable growth potential. The lower growth rate of toxic *M. aeruginosa* was probably caused by the excess energy cost for microcystin production [64]. Secondly, EPS production by toxic *M. aeruginosa* could provide a better adaptation to UV-B radiation. In this study, toxic *M. aeruginosa* produced more BEPS in the early stage in the UV-B treatment, when tryptophan-like substances in BEPS could absorb UV-B radiation and play the role of precursor to UV-absorbing metabolites [65]. The decrease of BEPS and increase of SEPS at the later stage could be explained as some UV-absorbing compounds were degraded, and this contributed to the decreased adaptation of algae to UV radiation under normal growth conditions [49,57]. Meanwhile, toxic *M. aeruginosa* excreted more SEPS, and organic matter in SEPS had a positive effect on algal aggregation [41,66]. The aggregated morphology of algal cells could be beneficial to reduce photo-induced damage by shading [67,68] and this was regarded as one kind of defense against UV-B in the natural waters. Furthermore, the high iron availability for algal cells could decrease UV-induced damages [27] and higher EPS could serve as an important iron reservoir that helps toxic *M. aeruginosa* to better cope with UV-B radiation [11,60].

Moreover, toxic *M. aeruginosa* exhibited a better antioxidant response in the UV-B treatment. In our study, two *M. aeruginosa* species could promote the synthesis of CAR and PC in the UV-B treatment, which was further enhanced with nutrient enrichment. As mentioned above, the beneficial effects of CAR and PC included the alleviation of photo-induced damage and the promotion of self-repair [15,69]. Moreover, microcystin synthesis by toxic *M. aeruginosa* could contribute to a higher fitness of cells under UV-B irradiation through a covalent interaction with the cysteine residue of proteins [70]. Consequently, *F_v_/F_m_* decline was lower and the recovery rate was faster for toxic *M. aeruginosa* under two different conditions. Xu et al. [18] also indicated that toxic *M. aeruginosa* had a competitive advantage relative to non-toxic strain in a changing light environment via stronger antioxidant capacity (higher SOD activity and the synthesis of microcystin) and quicker PSII recovery capacity The decrease of CAR and PC on Day 8 under normal growth conditions was related to the photooxidation and photodegradation of pigments, when the biological resources in the cultures might be not enough for the algal resynthesis of pigments and other efficient metabolic processes [71]. Compared to PAR treatment, the higher CAR/Chl-a and PC/Chl-a ratios of two *M. aeruginosa* species under normal growth conditions and higher CAR/Chl-a and PC/Chl-a ratios of all three species under nutrient enrichment conditions probably indicated their increased acclimation to prolonged UV-B exposure [13,72]. This was consistent with results obtained by Jiang et al. [50]. Although increased cell density partially reduced UV-B radiation at the later stage of incubation, our results could be mainly ascribed to the adaptation capability of algae to UV radiation in the 2-cm depth dishes.

### 4.3. Effects of Nutrient Enrichment and Algal Competition Characteristics

Whereas scholars have often studied the influences and mechanisms of UV radiation on algae, fewer studies have focused on the effects of nutrient enrichment. Meanwhile, the role of UV-B radiation in determining interspecific competition has not been clearly elucidated.

Combining the diurnal changes of algal *F_v_/F_m_* and algal growth patterns under different growth conditions, nutrient enrichment alleviated the negative effects of UV-B radiation on three species in our study. This was in accordance with our previous findings that higher P availability could enhance algal adaptation to UV radiation [26]. Zheng et al. [29] also reported that UV-induced inhibition of algal growth and photosynthetic production changed in accordance with the changes of the chemical environment in the water. In our study, the beneficial effects of nutrient enrichment also included decreasing algal sensitivity to UV-B radiation and increasing its self-repair efficiency. For example, higher contents of CAR and PC in cells with nutrient enrichment could help algae to counteract UV-induced damages [50,73], which resulted in lower ROS in cells and lower decline degrees of algal *F_v_/F_m_* on each day. Meanwhile, three species did not require a great deal of energy and biological resources to deal with UV-B radiation, and they could better promote their growth after self-repair with more nutrients in the medium, such as the photo-reactivation of DNA or resynthesis of D1 proteins [18,22]. Therefore, algal μ_max_ values were higher and three species persistently grew in the UV-B treatment under nutrient enrichment conditions. Since toxic *M. aeruginosa* exhibited a higher adaptation capability to UV-B radiation, as previously discussed, the beneficial effects of nutrient enrichment were best for toxic *M. aeruginosa*, and its growth was comparable between PAR and UV-B treatment during the whole incubation.

Wind-induced mixing of water and sediment resuspension could cause pulse fluctuations of irradiation conditions and high nutrient availability in the water, where different algal species coexist. Thus, the co-cultures under nutrient enrichment conditions might partly explain the competitive advantages of typical species in the field. Different from the mono-cultures, *C. pyrenoidosa* was not always the fastest-growing species in the co-cultures, and exposure to UV-B radiation could enhance the growth advantages of *M. aeruginosa*. Our previous study indicated that the augmentation of algal P quota could alleviate or eliminate the negative effects of UV radiation on algae [26]. Considering that *M. aeruginosa* had a faster and better P accumulation ability compared to other typical species in freshwater ecosystems [74], *M. aeruginosa* might have a stronger adaptation capability to UV-B radiation and a stronger competitive advantage in the co-cultures. However, since nutrients were not limited under nutrient enrichment conditions, allelopathy effects between species might have a more important role in the co-cultures [19,75]. In our study, two *M. aeruginosa* species demonstrated a greater inhibition effect on *C. pyrenoidosa* growth compared with the negative effects of *C. pyrenoidosa* on *M. aeruginosa*. For example, when the secondary metabolites of green algae showed declining inhibitory effects as incubation progressed, the extracts of cyanobacteria and microcystins were often more effective to inhibit the growth of other species [24,76]. Therefore, μ_max_ of *C. pyrenoidosa* decreased greatly and two *M. aeruginosa* species outcompeted *C. pyrenoidosa* at the later stage in the PAR treatment. Meanwhile, toxic *M. aeruginosa* showed a greater competitiveness to maintain high μ_max_ and inhibit *C. pyrenoidosa* growth in the co-cultures. As mentioned above, the higher EPS contents and microcystin of *M. aeruginosa* cells were conducive to the adaptation of *Microcystis* to UV-B radiation [11,70]. Furthermore, the aggregation of *Microcystis* might prevent *C. pyrenoidosa* to utilize PAR for self-repair or recovery after UV-B radiation [77]. Consequently, non-toxic and toxic *M. aeruginosa* were dominant from the beginning in the UV-B treatment, and toxic *M. aeruginosa* also had a greater impact in depressing the growth of *C. pyrenoidosa*. In this sense, the dominance of cyanobacteria and advantages of toxic *M. aeruginosa* could be enhanced in UV-radiated waters with severer eutrophication. However, the complexities and likely influence of coexisting yet unexamined factors deserve a further in situ study in the future.

## 5. Conclusions

(1) Compared with PAR, 4 h of ambient UV-B radiation could exert oxidative stresses and negative effects on the photosynthesis and growth of three algal species under normal growth conditions. The adopted UV- B treatment did not cause lethal effects on algae, and three species could grow with adaptive responses, including EPS production, regulation of SOD activity, synthesis of photosynthetic pigments, and *F_v_/F_m_* recovery.
(2) Three species exhibited strain-specific responses to ambient UV-B radiation in the mono-cultures, when toxic *M. aeruginosa* was more tolerant and showed a higher adaptation capability to UV-B, including lower sensitivity and better self-repair efficiency. In addition to stable μ_max_ in two treatments, higher production of EPS, and enhanced production of CAR and PC under UV-B radiation, toxic *M. aeruginosa* showed a better recovery of its photosynthetic efficiency.
(3) Nutrient enrichment could alleviate the negative effects of UV-B radiation on algae, and the growth of toxic *M. aeruginosa* was comparable between PAR and UV-B treatment. In the co-cultures with nutrient enrichment, *M. aeruginosa* gradually outcompeted *C. pyrenoidosa* in the PAR treatment, and UV-B treatment enhanced the growth advantages of *M. aeruginosa*, when toxic *M. aeruginosa* showed a greater competitiveness to maintain high μ_max_ and inhibit the growth of *C. pyrenoidosa*.

## Figures and Tables

**Table 1:** Contents of photosynthetic pigments of three species in the PAR and UV-B treatments on Day 1 and Day 8 under normal growth conditions).

Contents of Piments (pg/cell)		*C. pyrenoidosa*		Non-Toxic *M. aeruginosa*		Toxic *M. aeruginosa*	
		PAR	UV-B	PAR	UV-B	PAR	UV-B
Day 1	Chl-a	0.14 ± 0.02	0.15 ± 0.02	0.18 ± 0.03	0.16 ± 0.01	0.17 ± 0.02	0.17 ± 0.03
	CAR	0.12 ± 0.02	0.19 ± 0.02 *	0.07 ± 0.01	0.11 ± 0.01 *	0.06 ± 0.02	0.12 ± 0.02 *
	PC	\	\	0.62 ± 0.07	0.84 ± 0.06 *	0.55 ± 0.03	0.75 ± 0.04 *
	CAR/Chl-a	0.86 ± 0.02	1.25 ± 0.06 *	0.41 ± 0.02	0.68 ± 0.05 *	0.37 ± 0.05	0.72 ± 0.01 *
	PC/Chl-a	\	\	3.46 ± 0.15	5.15 ± 0.35 *	3.26 ± 0.16	4.64 ± 0.28 *
Day 8	Chl-a	0.36 ± 0.04	0.24 ± 0.03 *	0.27 ± 0.01	0.11 ± 0.01 *	0.25 ± 0.01	0.12 ± 0.02 *
	CAR	0.18 ± 0.01	0.09 ± 0.01 *	0.11 ± 0.01	0.07 ± 0.01 *	0.10 ± 0.02	**0.05 ± 0.01**
	PC	\	\	0.70 ± 0.01	0.54 ± 0.04 *	0.67 ± 0.03	0.46 ± 0.03 *
	CAR/Chl-a	0.50 ± 0.02	0.39 ± 0.03 *	0.42 ± 0.05	0.68 ± 0.02 *	0.42 ± 0.06	0.63 ± 0.03 *
	PC/Chl-a	\	\	2.61 ± 0.03	5.07 ± 0.38 *	2.68 ± 0.05	4.12 ± 0.24 *

* Bold values with * indicated significant higher contents in the UV-B treatment compared with PAR treatment at *p* < 0.05, while those bold and underlined values with * indicated significant lower contents in the UV-B treatment compared with PAR treatment at *p* < 0.05.

**Table 2:** Contents of photosynthetic pigments of three species in the PAR and UV-B treatments on Day 1 and Day 8 under nutrient enrichment conditions.

Contents of Piments (pg/cell)		*C. pyrenoidosa*		Non-Toxic *M. aeruginosa*		Toxic *M. aeruginosa*	
		PAR	UV-B	PAR	UV-B	PAR	UV-B
Day 1	Chl-a	0.14 ± 0.01	0.15 ± 0.02	0.18 ± 0.02	0.16 ± 0.02	0.17 ± 0.02	0.17 ± 0.02
	CAR	0.12 ± 0.02	0.23 ± 0.02 *	0.09 ± 0.01	0.14 ± 0.01 *	0.08 ± 0.01	0.15 ± 0.01 *
	PC	\	\	0.67 ± 0.02	0.97 ± 0.04 *	0.58 ± 0.02	0.94 ± 0.02 *
	CAR/Chl-a	0.84 ± 0.05	1.52 ± 0.08 *	0.41 ± 0.01	0.83 ± 0.02 *	0.37 ± 0.05	0.88 ± 0.09 *
	PC/Chl-a	\	\	3.57 ± 0.22	6.01 ± 0.48 *	3.17 ± 0.18	5.58 ± 0.42 *
Day 8	Chl-a	0.50 ± 0.02	0.35 ± 0.04 *	0.41 ± 0.02	0.30 ± 0.02 *	0.39 ± 0.01	0.29 ± 0.01 *
	CAR	0.23 ± 0.02	0.27 ± 0.01 *	0.18 ± 0.02	0.26 ± 0.01 *	0.15 ± 0.01	**0.19 ± 0.01**
	PC	\	\	0.70 ± 0.02	0.83 ± 0.03 *	0.69 ± 0.01	0.79 ± 0.03 *
	CAR/Chl-a	0.46 ± 0.03	0.78 ± 0.06 *	0.45 ± 0.05	0.86 ± 0.06 *	0.38 ± 0.02	0.68 ± 0.02 *
	PC/Chl-a	\	\	1.72 ± 0.02	3.83 ± 0.21 *	1.78 ± 0.09	2.84 ± 0.25 *

* Bold values with * indicated significant higher contents in the UV-B treatment compared with PAR treatment at *p* < 0.05, while those bold and underlined values with * indicated significant lower contents in the UV-B treatment compared with PAR treatment at *p* < 0.05.

## Data Availability

The data that support the findings of this study are available from the corresponding author, upon reasonable request.

## Supplementary Materials

The following supporting information can be downloaded at: https://www.mdpi.com/article/10.3390/ijerph19095485/s1, Figure S1. The spectral power distribution and weighted UV radiation of UV- B lamps (TL20W/01RS, Philips) used in the irradiation experiment. Figure S2. A schematic diagram of the irradiation experiments. Figure S3. Fluorescence EEM spectra for EPS produced by three species. Figure S4. Whole-cell absorption spectra of algal cultures at the beginning of mono-cultures. Cell cultures with OD_680_ of 0.10 were used for measurement and adsorption values were normalized to the optical density at OD_680_. Table S1. Composition of the modified BG_11_ medium under different growth conditions in our experiment. Table S2. The maximum growth rate (μ_max_, d^−1^) and maximum cell density (10^6^ cells/mL) of three species in the mono-cultures and co-cultures under nutrient enrichment conditions and the percentage change of maximum cell density showing in parentheses. References [57,78,79,80] are cited in the supplementary materials.
