# Supplementary material for: Comparative Study of Algal Responses and Adaptation Capability to Ultraviolet Radiation with Different Nutrient Regimes
Source: Int J Environ Res Public Health. 2022 Apr 30;19(9):5485. doi: 10.3390/ijerph19095485 (PMC9104955; doi:10.3390/ijerph19095485)
Supplement: Supplementary file 1 [file ijerph-19-05485-s001.zip › ijerph-1631771-supplementary.pdf]

Contents of Supplementary Materials

Measurement of ROS production by *M. aeruginosa* and the SOD activity

Measurement of the photosynthetic pigments in single cells

Figure S1. The spectral power distribution and weighted UV radiation of UV- B lamps (TL20W/01RS, Philips) used in the irradiation experiment.

Figure S2. A schematic diagram of the irradiation experiments.

Figure S3. Fluorescence EEM spectra for EPS produced by three species.

Figure S4. Whole-cell absorption spectra of algal cultures at the beginning of mono-cultures. Cell cultures with OD_{680} of 0.10 were used for measurement and adsorption values were normalized to the optical density at OD_{680}.

Table S1. Composition of the modified BG_{11} medium under different growth conditions in our experiment.

Table S2. The maximum growth rate (μ_{max}, d^{-1}) and maximum cell density (10^6 cells/mL) of three species in the mono-cultures and co-cultures under nutrient enrichment conditions and the percentage change of maximum cell density showing in parentheses.

ROS in algal cells and SOD activity

For the measurement of ROS in algal cells, cells of three species were regularly collected by centrifugation (5000 *g* for 10 min, 4°C), washed with phosphate-buffered saline (PBS, 50 mmol L^{-1}, pH 8.0), and suspended in the PBS solution buffer. Then, the production of reactive oxygen species (ROS) was monitored using the ROS sensitive fluorescence probe 2’ 7’-dichlorohydrofluorescein diacetates (DCFH-DA [1]). Cells were incubated with 10 μmol L^{-1} DCFH-DA (Sigma Aldrich, USA), which could diffuse into cells and its acetate groups were cleaved by intracellular esterases to produce 2′ 7′-dichlorohydrofluorescein (DCFH). Afterwards, intracellular ROS can oxidize DCFH to the highly fluorescent 2′ 7′-dichlorofluorescein (DCF), and the fluorescence intensity is proportional to the amount of ROS produced by algal cells. After incubation for 15 min at 37°C in the dark, cells were washed twice with PBS (pH 8.0) and the fluorescence intensity was measured with the excitation at 488 nm and the emission at 525 nm [2].

For the measurement of algal SOD activity, cells of three species were regularly collected by filtration using the 0.2-μm mixed cellulose ester filters (Whatman), which were then re-suspended in PBS solution (50 mmol L^{-1}, pH 8.0). The cells were disrupted by an ultrasonic cell pulverizer surrounded by ice bags. After centrifugation (5000 g for 10 min, 4°C), the supernatant was used for the detection of superoxide dismutase (SOD) activity with an Assay Kit (Jiancheng Bioengineering Institute, Nanjing, China).

Contents of photosynthetic pigments

At different stages of the incubation (Day 1 and 8) in the mono-cultures, a 10-mL subsample of algal cultures was collected and immediately filtrated through 0.22-μm GF/C filters (Whatman). The vacuum pressure was below 20 kPa to minimize the lysis and physical damage of algal cells. The filters with retained cells were extracted with a 90% acetone solution for 24-h in the dark and the absorbency of the supernatant was determined at 662, 645 and 470 nm using a UV-visible spectrophotometer, respectively. Then, chlorophyll a (Chl-*a*) and carotenoid (CAR) contents were calculated using the following equation [3].

$$\text{Chl-}a\ (\mu g/mL)=11.75*OD_{662}-2.35*OD_{645}$$

$$\text{CAR}\ (\mu g/mL)=\left(1000*OD_{470}-2.27*C\text{hl-}a-81.4*\left(18.61*OD_{645}-3.96*OD_{662}\right)\right)/227$$

Meanwhile, biliproteins in non-toxic and toxic *M. aeruginosa* cells were frozen repeatedly in liquid N_2 and thawed in a 0.05-mol L^{-1} phosphate buffer (pH 6.7) [2,4]. The homogenate solution was centrifuged at 4000 *g* for 15 min, and the absorbency of the supernatant was measured at 615 and 652 nm. Then, phycocyanin content (PC) was calculated using the following equation.

$$\text{PC}\ (\mu g/mL)=163.2*OD_{615}-117.1*OD_{650}$$

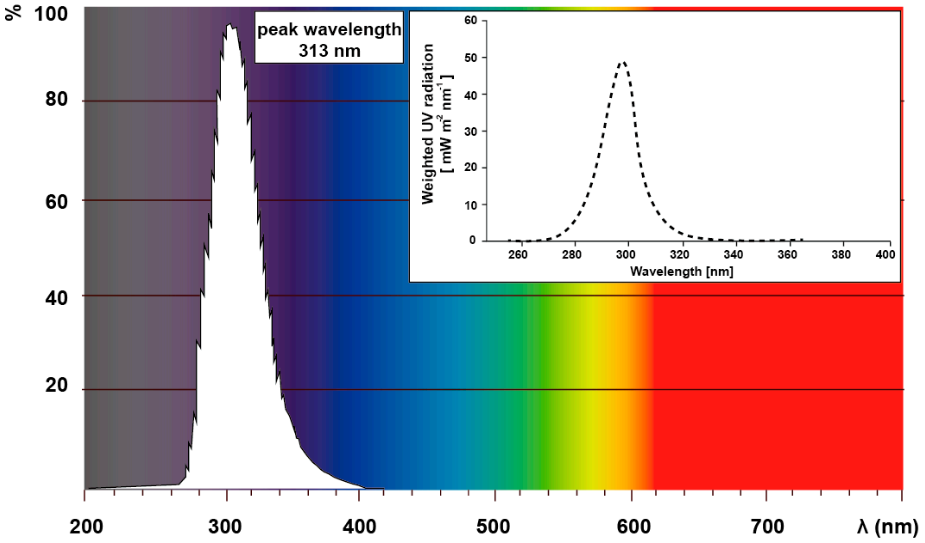

Figure S1. The spectral power distribution and weighted UV radiation of UV- B lamps (TL20W/01RS, Philips) used in the irradiation experiment.

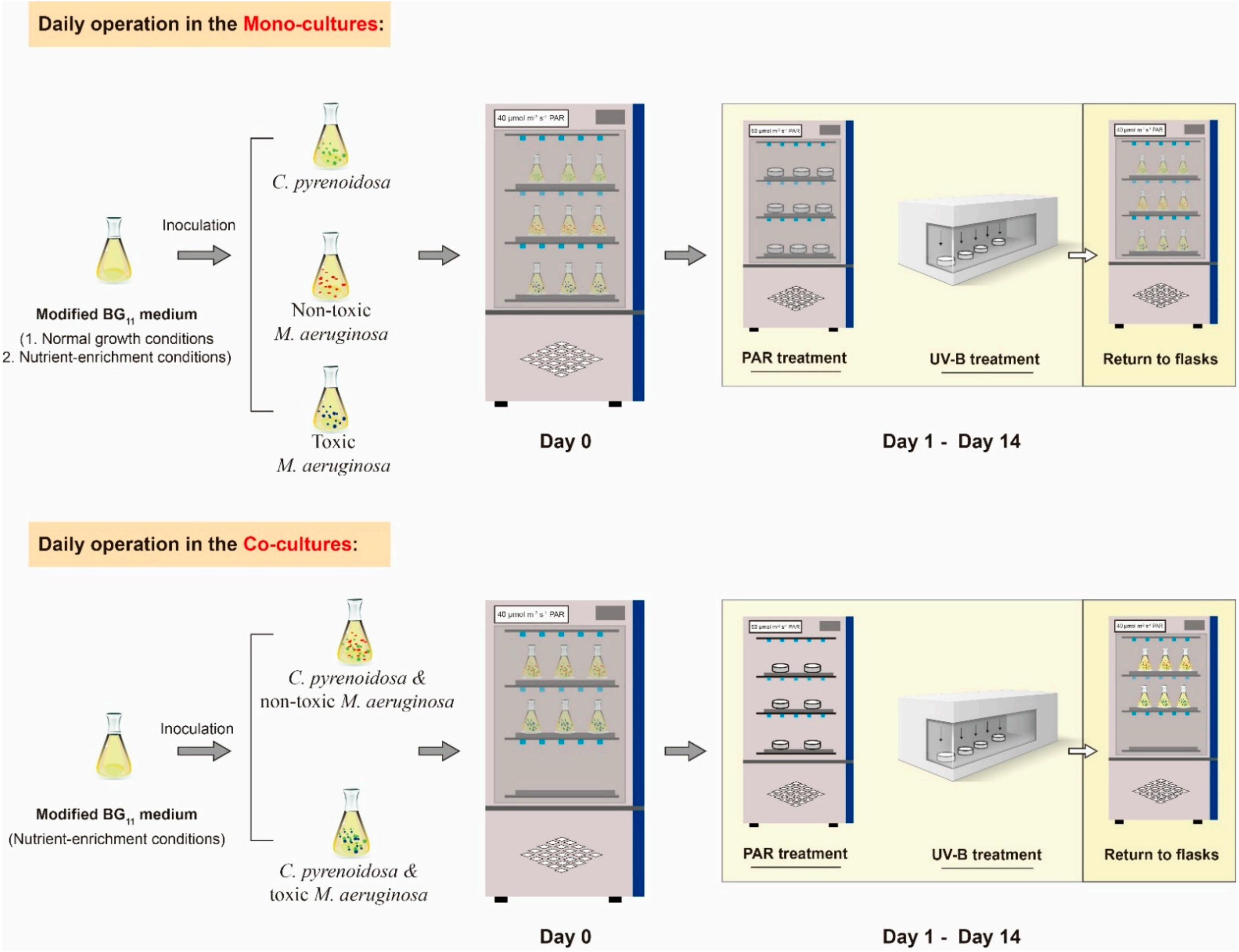

Figure S2. A schematic diagram of the irradiation experiments.

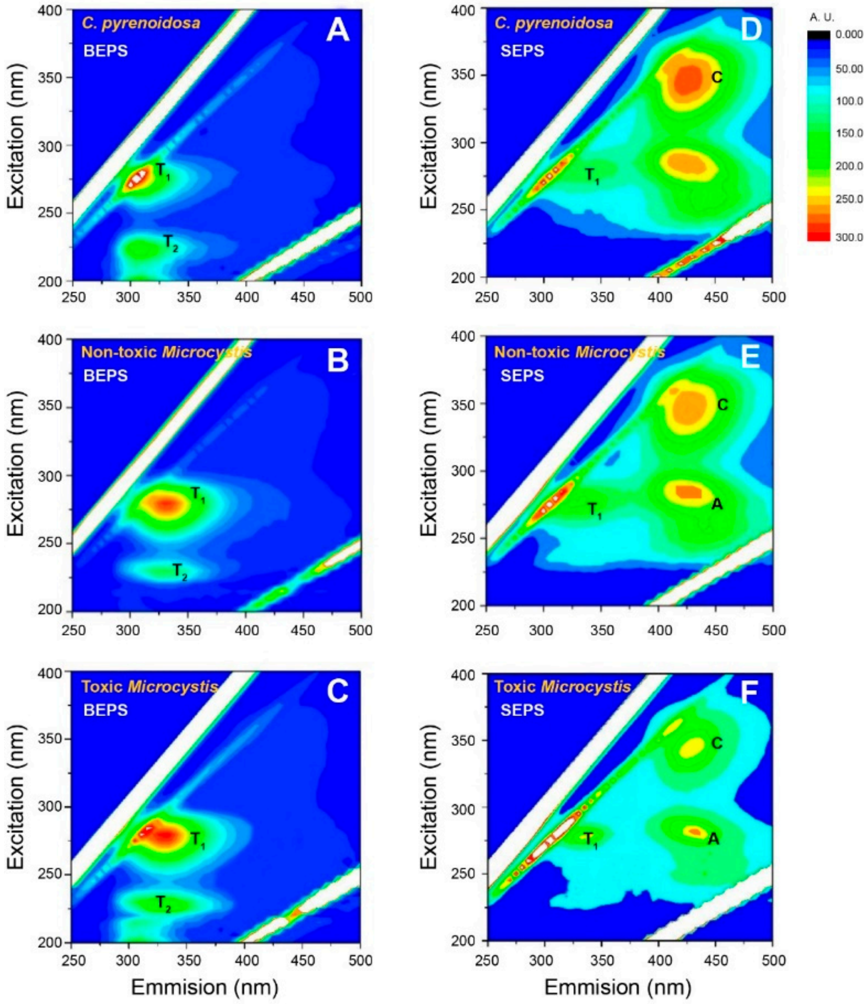

Figure S3. Fluorescence EEM spectra for EPS produced by three species.

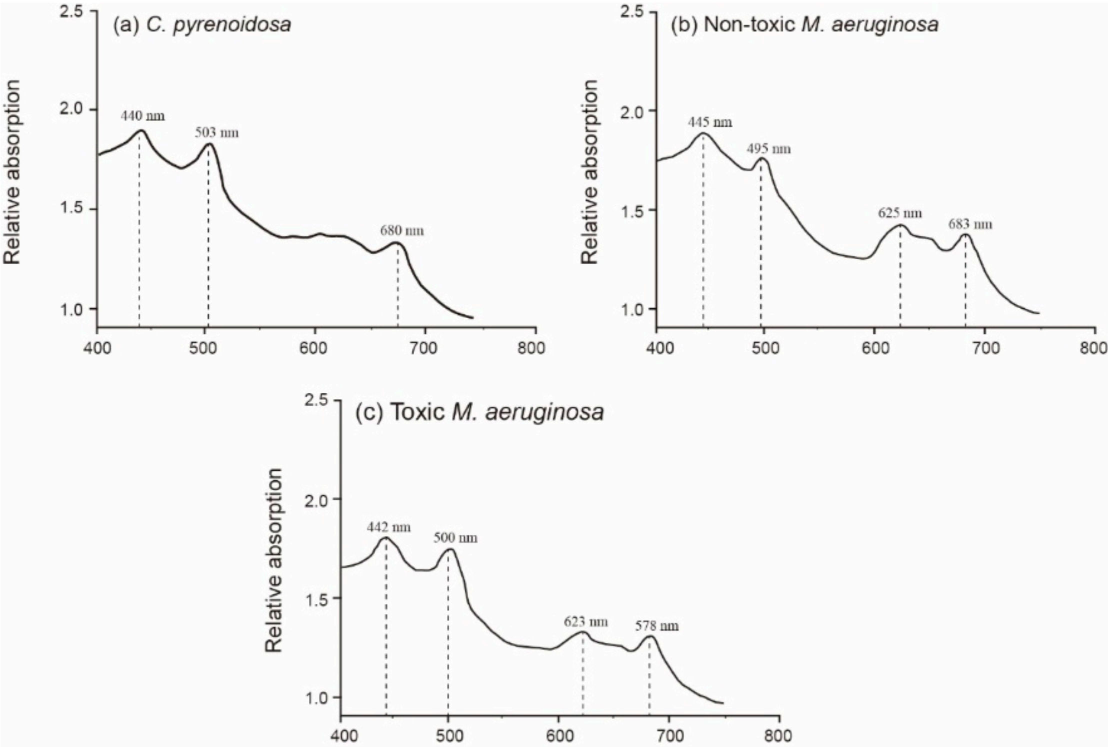

Figure S4. Whole-cell absorption spectra of algal cultures at the beginning of mono-cultures. Cell cultures with OD_{680} of 0.10 were used for measurement and adsorption values were normalized to the optical density at OD_{680}.

Table S1. Composition of the modified BG_{11} medium under different growth conditions in our experiment.

Stock solution		Normal growth conditions		Nutrient enrichment conditions	
Composition	Concentration	Dosage [/L culture medium]	Concentration	Dosage [/L culture medium]	Concentration
$K_2HPO_4{\cdot}3H_2O$	0.175 mol/L	0.5 mL	0.088 μmol/L	1 mL	0.175 μmol/L
$MgSO_4{\cdot}7H_2O$	75 mg/mL	1 mL	37.5 mg/L	1 mL	75 mg/L
$CaCl_2{\cdot}2H_2O$	36 mg/mL	1 mL	18 mg/L	1 mL	36 mg/L
Citric Acid	6 mg/mL	1 mL	3 mg/L	1 mL	6 mg/L
Ammonium Ferric Citrate	20 mg/mL	0.5 mL	10 mg/L	1.5 mL	30 mg/L
EDTA-Na_2	1 mg/mL	1 mL	0.5 mg/L	1 mL	1 mg/L
Na_2CO_3	20 mg/mL	1 mL	10 mg/L	1 mL	20 mg/L
$NaNO_3$	2.35 mol/L	1.5 mL	3.53 μmol/L	3 mL	7.06 μmol/L
A_5 solution:		**N/P ratio**	40.11	**N/P ratio**	40.11
H_3BO_3	2.86 g/L	1 mL	\	1 mL	\
$MnCl_2{\cdot}4H_2O$	1.86 g/L				
$ZnSO_4{\cdot}7H_2O$	0.22 g/L				
$CuSO_4{\cdot}5H_2O$	80 mg/L				
$(NH_4)_2MoO_4{\cdot}3H_2O$	0.39 g/L				
$Co(NO_3)_2{\cdot}6H_2O$	0.05 g/L				

Table S2. The maximum growth rate (μ_{max}, d^{-1}) and maximum cell density (10^6 cells/mL) of three species in the mono-cultures and co-cultures under nutrient enrichment conditions and the percentage change of maximum cell density showing in parentheses.

Algal species in different cultures		PAR treatment		UV-B treatment	
		μ_{max}	Maximum density	μ_{max}	Maximum density
Mono-cultures	*C. pyrenoidosa*	1.68	24.15	1.17	14.38
	Non-toxic *M. aeruginosa*	0.86	17.22	0.83	13.12
	Toxic *M. aeruginosa*	0.76	15.58	0.74	13.54
Co-cultures	*C. pyrenoidosa*	1.12	13.25 (54.9%)	0.67	6.05 (42.1%)
	Non-toxic *M. aeruginosa*	0.82	12.21 (70.9%)	0.78	9.84 (75.0%)
	C. pyrenoidosa	0.92	7.55 (31.3%)	0.54	4.52 (31.4%)
	Toxic *M. aeruginosa*	0.81	11.34 (72.8%)	0.78	10.34 (76.4%)

References

1. He, Y.Y.; Häder, D.P. Involvement of reactive oxygen species in the UV-B damage to the cyanobacterium *Anabaena* sp. *J. Photoch. Photobiol. B*. **2002**, *66*, 73–80.
2. Zhang, Y.; Jiang, H.B.; Qiu, B.S. Effects of UVB Radiation on competition between the bloom-forming cyanobacterium *Microcystis aeruginosa* and the chlorophyceae *Chlamydomonas microsphaera*. *J. Phycol.* **2013**, *49*, 318.
3. Takaichi, S.; Mochimaru, M. Carotenoids and carotenogenesis in cyanobacteria: unique ketocarotenoids and carotenoid glycosides. *Cell. Mol. Life Sci.* **2007**, *64*, 2607–2619.
4. Lüder, U.H.; Knoetzel, J.; Wiencke, C. Acclimation of photosynthesis and pigments to seasonally changing light conditions in the endemic Antarctic red macroalga *Palmaria decipiens*. *Polar Biol.* **2001**, *24*, 598–603.
